# Modeling Surface-Enhanced Spectroscopy With Perturbation Theory

**DOI:** 10.3389/fchem.2019.00470

**Published:** 2019-07-16

**Authors:** Niclas S. Mueller, Stephanie Reich

**Affiliations:** Department of Physics, Freie Universität Berlin, Berlin, Germany

**Keywords:** surface-enhanced Raman spectroscopy (SERS), plasmonics, perturbation theory, second quantization formalism, optical interference

## Abstract

Theoretical modeling of surface-enhanced Raman scattering (SERS) is of central importance for unraveling the interplay of underlying processes and a predictive design of SERS substrates. In this work we model the plasmonic enhancement mechanism of SERS with perturbation theory. We consider the excitation of plasmonic modes as an integral part of the Raman process and model SERS as higher-order Raman scattering. Additional resonances appear in the Raman cross section which correspond to the excitation of plasmons at the wavelengths of the incident and the Raman-scattered light. The analytic expression for the Raman cross section can be used to explain the outcome of resonance Raman measurements on SERS analytes as we demonstrate by comparison to experimental data. We also implement the theory to calculate the optical absorption cross section of plasmonic nanoparticles. From a comparison to experimental cross sections, we show that the coupling matrix elements need to be renormalized by a factor that accounts for the depolarization by the bound electrons and interband transitions in order to obtain the correct magnitude. With model calculations we demonstrate that interference of different scattering channels is key to understand the excitation energy dependence of the SERS enhancement for enhancement factors below 10^3^.

## 1. Introduction

Surface-enhanced Raman scattering (SERS) is the giant increase in the Raman cross section of a molecule close to a metallic nanostructure (Fleischmann et al., 1974). The local enhancement can exceed ten orders of magnitude making SERS an ideal tool for analytical chemistry that can be even applied for single-molecule detection (Kneipp et al., 1997; Nie and Emory, 1997; Sharma et al., 2012; Wang et al., 2013). The enhancement arises from an interplay of several mechanisms that act simultaneously. There is a general agreement that the strongest enhancement mechanism is the excitation of localized surface plasmon resonances in noble metal nanostructures (Ru and Etchegoin, 2009; Ding et al., 2017). The collective oscillation of conduction electrons leads to intense electromagnetic near fields close to the metal surface that drive the Raman process. The largest enhancement arises from so-called electromagnetic hot spots that occur in the nanometer gaps between plasmonic nanoparticles with a local field intensity that can be five orders of magnitude larger than that of the incident light. Besides plasmonic enhancement, the SERS intensity is affected by chemical enhancement which encompasses a number of effects that concern the chemical interaction of the molecule with the metal surface (Jensen et al., 2008). The metal-molecule interaction can lead to hybridized or charge transfer states that introduce new resonances in the SERS cross section or the molecular resonances can be shifted which may lead to an increase or decrease of the SERS intensity (Osawa et al., 1994; Morton and Jensen, 2009; Darby et al., 2015; Hu et al., 2015; Sevinc et al., 2016). A successful design of SERS substrates depends critically on our understanding of the underlying enhancement mechanisms and a predictive theoretical modeling.

Even though the phenomenon of SERS was discovered more than 40 years ago there are many aspects that remain not fully understood (Moskovits, 2013). Due to the multitude of involved processes it is a challenge to predict the outcome of a SERS experiment, such as the magnitude of the enhancement and its excitation energy dependence. For many years the focus has been on developing microscopic theories for the chemical enhancement mechanism which give insight into the interaction of a molecule with a metal surface and its effect on the Raman spectrum (Jensen et al., 2008; Lombardi and Birke, 2008; Galperin et al., 2009; Hu et al., 2015). The plasmonic enhancement mechanism, on the other hand, is usually modeled with a purely electromagnetic enhancement factor, which is known as the theory of electromagnetic enhancement (Ru and Etchegoin, 2009; Ding et al., 2017). This macroscopic approach is a powerful tool for designing SERS substrates with large enhancement factors but lacks microscopic insight into the different scattering processes underlying SERS. Recently, there has been renewed interest in plasmonic enhancement and in developing microscopic theories that complement the EM enhancement model and expand it to include quantum mechanical effects, such as electron tunneling, optomechanical backaction and non locality (Pustovit and Shahbazyan, 2006; Davis et al., 2010; Roelli et al., 2016; Schmidt et al., 2016; Kamandar Dezfouli and Hughes, 2017; Neuman et al., 2018).

Based on the microscopic theory of Raman scattering we suggested to describe SERS as higher-order Raman (HORa) scattering and developed a theory that treats the plasmonic excitation as a part of the Raman process (Mueller et al., 2016). The localized surface plasmon resonances were included in the Raman cross section in the same way as the molecular resonances. Considering the excitations of the plasmon and of the molecular transitions as subsequent steps of the Raman process allowed us to derive selection rules for SERS with group theory (Jorio et al., 2017). On the experimental side, we designed SERS substrates that allowed to measure exclusively the plasmonic enhancement of the Raman cross section (Heeg et al., 2013, 2014; Mueller et al., 2017b; Wasserroth et al., 2018). The approach of describing SERS as higher-order Raman scattering gives an intuitive picture of what happens in the various steps of the Raman transition (absorption of light by plasmon, electronic excitation by the plasmonic near field, vibronic coupling and so forth). We therefore argued that it should be an excellent tool to fit experimental data and extract information like the strength of light-matter coupling and the energy of the plasmonic resonance, but have not performed such an analysis.

Here we revisit the theory of SERS as higher-order Raman scattering and draw the comparison to experiments. We discuss how the analytic expression for the SERS cross section is used to interpret the excitation energy dependence of the enhancement in experiments. By treating the localized surface plasmon as a quasi-particle we derive analytic expressions for the coupling matrix elements. We account for the depolarization by bound electrons and interband transitions in the interaction Hamiltonians. These contributions were omitted in our previous work that, therefore, overestimated SERS enhancement factors. We calculate the optical absorption cross section of gold and silver nanoparticles as intermediate steps in the Raman process. The excellent agreement with experiments supports the quantitative predictions of our theory. Based on model calculations for a molecule close to a silver nanoparticle we demonstrate that interference between different scattering processes can strongly affect the excitation energy dependence of the SERS enhancement. Our theory leads to the same expression for the plasmonic enhancement in SERS as the commonly used electromagnetic enhancement. In addition, it is used to extract experimental data on the plasmonic system from Raman spectroscopy without requiring a detailed knowledge of the geometry of the plasmonic nanostructure as an input parameter.

## 2. SERS as Higher-Order Raman Scattering

The theory of surface-enhanced Raman scattering as a higher-order Raman process was introduced in Mueller et al. (2016) and will be reviewed in this section in order to set a theoretical basis for the rest of this paper. The implementation is based on the microscopic theory of Raman scattering which uses perturbation theory to calculate the Raman scattering cross section (Long, 2002; Yu and Cardona, 2010). The main idea is to consider the plasmonic excitation, similar to the molecular excitation, as a step in the Raman scattering process. SERS is therefore described as a higher-order Raman process and the plasmonic resonances appear in the Raman cross section.

**Figure 1:**
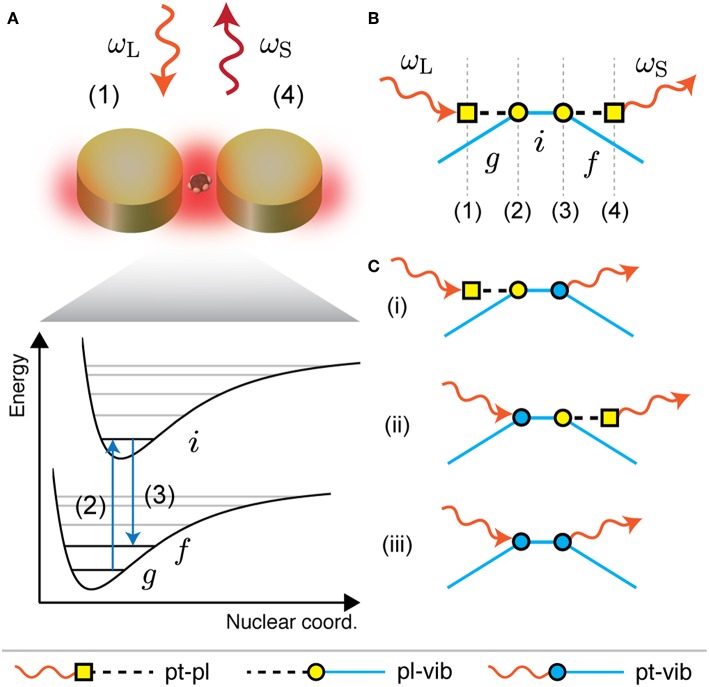
SERS as higher-order Raman scattering. **(A)** Sketch of a plasmon-enhanced Raman process relevant for SERS. The steps are: (1) excitation of a plasmon by the incoming light ω_L_; (2) molecular transition from ground state *g* to intermediate state *i* by coupling to the plasmonic near field; (3) molecular relaxation to final state *f* and excitation of the plasmon; (4) emission of Raman-scattered light ω_S_ by the plasmon. **(B)** Plasmon-enhanced Raman process in **(A)** illustrated as a Feynman diagram which corresponds to fourth-order perturbation theory. **(C)** Feynman diagrams of three other scattering processes that are relevant for SERS, i.e., (i) only the incoming light or (ii) only the Raman-scattered light couples to the plasmon and (iii) the Raman process without plasmonic enhancement.

We consider a plasmon-enhanced Raman process as illustrated in Figure 1A that consists of the following steps: (1) The incoming laser light ω_L_ excites a localized surface plasmon ω_pl_. (2) The plasmonic nanostructure couples via its optical near field to a nearby molecule and induces a transition from the vibronic ground state *g* to an intermediate state *i*. The intermediate state can be also a virtual state. (3) The molecule relaxes to a final vibronic state *f* and excites again the localized surface plasmon. (4) Finally, the Raman-scattered light ω_S_ is emitted by the plasmonic nanostructure (Mack et al., 2017; Raab et al., 2017). The plasmon-enhanced Raman process can be also illustrated by the Feynman diagram in Figure 1B. Each vertex of the diagram corresponds to one of the four steps of the Raman process. Additionally, there are three other relevant scattering processes that take place simultaneously; see Figure 1C: The processes where only the incoming light (i) or only the Raman-scattered light (ii) couples to the localized surface plasmon and the Raman process without plasmonic enhancement (iii).

Following Martin and Falicov (1975) and Yu and Cardona (2010) the Feynman diagrams can be translated into Raman scattering amplitudes with perturbation theory.

(1) $$\begin{array}{l} K_{\text{pl}-\text{pl}}^{w,w^{\prime },i}\left(\omega_{\text{L}}\right)\\ \;=\frac{M_{\text{pt}-\text{pl}}^{w^{\prime }}M_{\text{pl}-\text{vib}}^{w^{\prime },i}M_{\text{vib}-\text{pl}}^{w,i}M_{\text{pl}-\text{pt}}^{w}}{\hbar^{3}\left(\omega_{\text{L}}-\omega_{f}^{\text{vib}}-\omega_{w^{\prime }}-\text{i}\gamma_{w^{\prime }}\right)\left(\omega_{\text{L}}-\omega_{i}^{\text{vib}}-\text{i}\gamma_{i}^{\text{vib}}\right)\left(\omega_{\text{L}}-\omega_{w}-\text{i}\gamma_{w}\right)} \end{array}$$

corresponds to the Feynman diagram in Figure 1B,

(2) $$\begin{array}{r} K_{\text{pl}-\text{pt}}^{w,i}\left(\omega_{\text{L}}\right)=\frac{M_{\text{pt}-\text{vib}}^{i}M_{\text{vib}-\text{pl}}^{w,i}M_{\text{pl}-\text{pt}}^{w}}{\hbar^{2}\left(\omega_{\text{L}}-\omega_{i}^{\text{vib}}-\text{i}\gamma_{i}^{\text{vib}}\right)\left(\omega_{\text{L}}-\omega_{w}-\text{i}\gamma_{w}\right)} \end{array}$$

to Figure 1C (i),

(3) $$\begin{array}{r} K_{\text{pt}-\text{pl}}^{w,i}\left(\omega_{\text{L}}\right)=\frac{M_{\text{pt}-\text{pl}}^{w}M_{\text{pl}-\text{vib}}^{w,i}M_{\text{vib}-\text{pt}}^{i}}{\hbar^{2}\left(\omega_{\text{L}}-\omega_{f}^{\text{vib}}-\omega_{w}-\text{i}\gamma_{w}\right)\left(\omega_{\text{L}}-\omega_{i}^{\text{vib}}-\text{i}\gamma_{i}^{\text{vib}}\right)} \end{array}$$

to Figure 1C (ii) and

(4) $$\begin{array}{r} K_{\text{pt}-\text{pt}}^{i}\left(\omega_{\text{L}}\right)=\frac{M_{\text{pt}-\text{vib}}^{i}M_{\text{vib}-\text{pt}}^{i}}{\hbar \left(\omega_{\text{L}}-\omega_{i}^{\text{vib}}-\text{i}\gamma_{i}^{\text{vib}}\right)} \end{array}$$

to Figure 1C (iii). $\hbar \omega_{i}^{\text{vib}}$ and $\hbar \omega_{f}^{\text{vib}}$ are the energies of the vibronic molecular states and ℏω_*w*_ and $\hbar \omega_{w^{\prime }}$ are the energies of two plasmon modes *w* and *w*′. The energy of the molecular ground state $\hbar \omega_{g}^{\text{vib}}$ was referenced to zero. $\gamma_{i}^{\text{vib}}=\hbar /\tau_{i}^{\text{vib}}$, γ_*w*_ = ℏ/τ_*w*_ and $\gamma_{w^{\prime }}=\hbar /\tau_{w^{\prime }}$ are the respective inverse life times $\tau_{i}^{\text{vib}}$, τ_*w*_ and $\tau_{w^{\prime }}$ of the excitations. The matrix elements $M_{i-j}$ correspond to the vertices of the Feynman diagrams and describe the coupling strength of the photon-plasmon (pt-pl), plasmon-molecule (pl-vib) and photon-molecule (pt-vib) interactions. We will derive explicit expressions below.

The energy terms in the denominators correspond to plasmonic and molecular resonances and generate the excitation energy dependence of the Raman cross section. When the incoming light matches the energy ℏω_*w*_ of a plasmon mode, the real part of the corresponding energy term vanishes which leads to a resonance of the Raman cross section with spectral width 2γ_*w*_. In the following we will term this “incoming plasmonic Raman resonance” because the incoming light matches a plasmon mode. Similarly an outgoing plasmonic Raman resonance occurs for $\omega_{\text{L}}-\omega_{f}^{\text{vib}}=\omega_{w^{\prime }}$, i.e., when the energy of the Raman-scattered light matches that of the plasmon mode. Furthermore, a molecular Raman resonance occurs when the incoming light matches a vibronic state *i*. The excitation of a virtual state is described by the off-resonant excitation of the vibronic state *i*. The resonances will have Lorentzian line shape. We will discuss the applicability of this approximation for plasmonic excitations below.

The Raman scattering rate that is relevant for the intensity that arrives at the detector can be calculated with the Fermi Golden Rule as

(5) $$\begin{array}{r} \Gamma \left(\omega_{\text{L}}\right)=\underset{f}{\sum }|\underset{w,w^{\prime },i}{\sum }K_{\text{SERS}}^{w,w^{\prime },i}\left(\omega_{\text{L}}\right){|}^{2}\frac{2\gamma_{\text{vib}}}{\hbar^{2}\left[{\left(\omega_{\text{L}}-\omega_{f}^{\text{vib}}\right)}^{2}+{\left(\gamma_{f}^{\text{vib}}\right)}^{2}\right]}, \end{array}$$

where $K_{\text{SERS}}^{w,w^{\prime },i}=K_{\text{pl}-\text{pl}}^{w,w^{\prime },i}+K_{\text{pl}-\text{pt}}^{w,i}+K_{\text{pt}-\text{pl}}^{w,i}+K_{\text{pt}-\text{pt}}^{i}$. $\gamma_{f}^{\text{vib}}$ is the inverse lifetime of the final vibronic state and corresponds to the spectral width $2\gamma_{f}^{\text{vib}}$ of the vibrational mode in the Raman spectrum. All Raman amplitudes that lead to the same final state *f* are summed before calculating the absolute square; i.e., summation over *w*, *w*′ and *i* in Equation (5). The different scattering channels might interfere constructively or destructively which will be discussed below. In a SERS experiment one typically divides the measured intensity of a Raman mode by a reference to calculate an enhancement factor

(6) $$\begin{array}{r} \text{EF}\left(\omega_{\text{L}}\right)=\frac{|\sum_{w,w^{\prime },i}K_{\text{SERS}}^{w,w^{\prime },i}\left(\omega_{\text{L}}\right){|}^{2}}{|\sum_{i}K_{\text{ref}}^{i}\left(\omega_{\text{L}}\right){|}^{2}}, \end{array}$$

where $K_{\text{ref}}^{i}\left(\omega_{\text{L}}\right)$ is given by an expression similar to Equation (4). The enhancement factor can be only written in this way when referencing to the intensity of the same Raman mode. In experiments the measured Raman intensity of the SERS analyte is typically divided by the intensity of the same analyte in solution (Le Ru and Etchegoin, 2013). The different dielectric environment might shift the molecular resonance of the SERS analyte with respect to that of the reference. In this case the energies of the intermediate molecular states $\hbar \omega_{i}^{\text{vib}}$ that appear in *K*_SERS_ and *K*_ref_ are different. On the other hand, if the molecular states of the SERS analyte and the reference are identical all terms related to the molecular resonance cancel and Equation (6) simplifies to

(7) $$\begin{array}{l} \text{EF}\left(\omega_{\text{L}}\right)=|\frac{{\tilde{M}}_{1}{\tilde{M}}_{2}}{\hbar^{2}\left(\omega_{\text{L}}-\omega_{\text{vib}}-\omega_{\text{pl}}-\text{i}\gamma_{\text{pl}}\right)\left(\omega_{\text{L}}-\omega_{\text{pl}}-\text{i}\gamma_{\text{pl}}\right)}\\ \;+\frac{\tilde{M_{1}}}{\hbar \left(\omega_{\text{L}}-\omega_{\text{pl}}-\text{i}\gamma_{\text{pl}}\right)}\\ \;+\frac{\tilde{M_{2}}}{\hbar \left(\omega_{\text{L}}-\omega_{\text{vib}}-\omega_{\text{pl}}-\text{i}\gamma_{\text{pl}}\right)}+1{|}^{2}, \end{array}$$

where ${\tilde{M}}_{1}$ and ${\tilde{M}}_{2}$ are coupling factors that describe the strength of the incoming and the outgoing plasmonic Raman resonances (Mueller et al., 2016). Additionally we have assumed that only one plasmon mode ω_pl_ is excited and set $\omega_{\text{vib}}\equiv \omega_{f}^{\text{vib}}$. Equation (7) is a purely plasmonic enhancement factor of the SERS cross section. The enhancement at the incoming plasmonic Raman resonance is

(8) $$\begin{array}{r} \text{EF}\left(\omega_{\text{pl}}\right)=\frac{\left({\tilde{M}}_{1}^{2}+\hbar^{2}\gamma_{\text{pl}}^{2}\right)\left[{\tilde{M}}_{2}^{2}-2\;\hbar \omega_{\text{vib}}{\tilde{M}}_{2}+\hbar^{2}\left(\omega_{\text{vib}}^{2}+\gamma_{\text{pl}}^{2}\right)\right]}{\hbar^{4}\gamma_{\text{pl}}^{2}\left(\omega_{\text{vib}}^{2}+\gamma_{\text{pl}}^{2}\right)} \end{array}$$

and the enhancement at the outgoing plasmonic Raman resonance is

(9) $$\begin{array}{r} EF\left(\omega_{\text{pl}}+\omega_{\text{vib}}\right)=\frac{\left({\tilde{M}}_{2}^{2}+\hbar^{2}\gamma_{\text{pl}}^{2}\right)\left[{\tilde{M}}_{1}^{2}+2\hbar \omega_{\text{vib}}{\tilde{M}}_{1}+\hbar^{2}\left(\omega_{\text{vib}}^{2}+\gamma_{\text{pl}}^{2}\right)\right]}{\hbar^{4}\gamma_{\text{pl}}^{2}\left(\omega_{\text{vib}}^{2}+\gamma_{\text{pl}}^{2}\right)}. \end{array}$$

We will demonstrate below that the enhancement at the incoming- and outgoing plasmonic Raman resonances can differ significantly because of inteference between different scattering channels.

**Figure 2:**
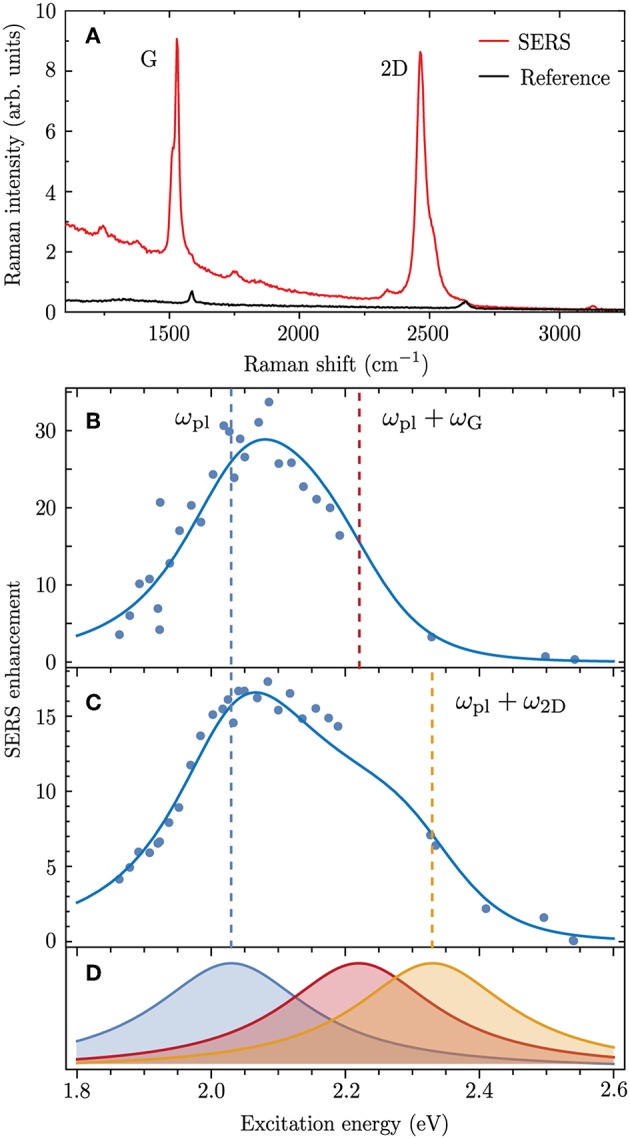
**(A)** SERS spectrum of graphene deposited on top of a gold nanodimer (red) and reference spectrum without the gold nanodimer (black) for ℏω_L_ = 1.94 eV. **(B,C)** Excitation energy dependence of the SERS enhancement measured for **(B)** the Raman G mode and **(C)** the 2D mode (data from Wasserroth et al., 2018). Solid lines are a fit to the experimental data with Equation (7) using ℏω_pl_ = 2.03 eV, ℏγ_pl_ = 140 meV and ${\tilde{M}}_{1}={\tilde{M}}_{2}=-1.5$ eV for the G mode and ${\tilde{M}}_{1}={\tilde{M}}_{2}=-1.52$ eV for the 2D mode. The Raman shifts ℏω_G_ = 0.19 eV and ℏω_2D_ ≈ 0.3 eV were obtained from the experimental spectra. The incoming (ω_L_ = ω_pl_) and outgoing ($\omega_{\text{L}}=\omega_{\text{pl}}+\omega_{f}^{\text{vib}}$) plasmonic Raman resonances are indicated as dashed lines. The enhancement factor in Equation (7) was divided by a factor of 230 as it gives a local enhancement while the experimental data are a global enhancement (see Wasserroth et al., 2018 for details). **(D)** Normalized incoming (blue) and outgoing plasmonic Raman resonances (red for G mode and orange for 2D mode) which constitute the resonance profiles in **(B,C)**.

In previous works we have designed SERS experiments which allow to extract the plasmonic enhancement of the SERS cross section (Heeg et al., 2013, 2014; Mueller et al., 2017b; Wasserroth et al., 2018). For this we used carbon nanostructures as SERS analytes, i.e., graphene, carbon nanotubes and carbon nanotubes filled with molecules. These structures have a Raman response that is strong enough to be detected in the absence of plasmonic enhancement. The experiments were designed in such a way that the Raman intensities with and without the plasmonic nanostructure could be compared directly. In Figure 2 we show the excitation-energy dependent SERS enhancement for graphene deposited on top of a gold nanodimer (Wasserroth et al., 2018). The enhancement was measured for the two prominent Raman modes of graphene, the carbon-carbon stretching G mode (ℏω_G_ = 0.19 eV) and the overtone of the ring-breathing mode 2D (ℏω_2D_ ≈ 0.3 eV). An exemplary SERS spectrum (red) and a reference spectrum recorded away from the plasmonic nanodimer (black) are shown in Figure 2A. The enhanced Raman modes are shifted with respect to the reference because of strain that is induced in the graphene lattice by the nanodimer (Mueller et al., 2017a). By using tunable laser excitation we measured the excitation energy dependence of the plasmonic enhancement which is plotted for the G mode in Figure 2B and for the 2D mode in Figure 2C.

The excitation energy dependence of the SERS enhancement can be interpreted with Equation (7) using ${\tilde{M}}_{1}$, ${\tilde{M}}_{2}$, ω_pl_ and γ_pl_ as fitting parameters. The fits are illustrated as solid lines in Figures 2B,C and match excellently the experimental data. The corresponding incoming and outgoing plasmonic Raman resonances are illustrated in Figure 2D. The excitation energy dependence of the SERS enhancement is narrower than the sum of the two resonances as it mainly arises from their product. An asymmetry appears because of interference between different scattering channels, which will be discussed in detail below. Generally we observed that the outgoing Raman resonance is weaker than the incoming Raman resonance.

## 3. Interaction Hamiltonians

The description of surface-enhanced Raman scattering as higher-order Raman scattering considers the excitation of the localized surface plasmon by the incoming light and the coupling of the plasmon to the molecule as two subsequent steps of the Raman process. This requires to treat the localized surface plasmon as a quasiparticle. In the following we derive analytic expressions for the coupling matrix elements based on a quantization of the localized surface plasmon. We use the same interaction Hamiltonians to describe the incoming scattering pathway (absorption of light by plasmon, excitation of vibronic transition in molecule by plasmonic near field) and the outgoing scattering pathway (excitation of plasmon by molecule, emission of Raman scattered light by plasmonic nanostructure). We demonstrated in Mueller et al. (2016) that this is a good approximation in the backscattering geometry; see also Ausman and Schatz (2009) and Ausman and Schatz (2012).

### 3.1. Quantization of the Localized Surface Plasmon

To quantize the localized surface plasmon resonances of a metallic nanoparticle, we use a jellium model which assumes a density N of free electrons that move in the presence of a uniform positively charged background (Gerchikov et al., 2002; Weick et al., 2005; Finazzi and Ciccacci, 2012). The center-of-mass Hamiltonian describing the collective motion of the electrons is given by

(10) $$\begin{array}{r} {\hat{H}}_{\text{pl}}=\frac{NV_{\text{p}}}{2}\underset{w}{\sum }\left(\frac{1}{m}{\hat{\Pi }}_{w}^{2}+m\omega_{w}^{2}{\hat{\text{Ψ}}}_{w}^{2}\right), \end{array}$$

where *V*_p_ is the volume of the metallic nanoparticle. ${\hat{\text{Ψ}}}_{w}$ and ${\hat{\Pi }}_{w}$ are canonical position and momentum coordinates that are written in terms of creation and annihilation operators ${\hat{b}}_{w}^{†}$ and ${\hat{b}}_{w}$ of a plasmon mode *w* as

(11) $$\begin{array}{r} {\hat{\text{Ψ}}}_{w}=\sqrt{\frac{\hbar }{2mNV_{\text{p}}\omega_{w}}}\left({\hat{b}}_{w}^{†}+{\hat{b}}_{w}\right) \end{array}$$

and

(12) $$\begin{array}{r} {\hat{\Pi }}_{w}=\text{i}\sqrt{\frac{m\;\hbar \omega_{w}}{2NV_{\text{p}}}}\left({\hat{b}}_{w}^{†}-{\hat{b}}_{w}\right). \end{array}$$

Using the bosonic commutation relations

(13) $$\begin{array}{r} \left[{\hat{b}}_{w},{\hat{b}}_{w^{\prime }}^{†}\right]=\delta_{w,w^{\prime }} \end{array}$$

the Hamiltonian in Equation (10) can be rewritten as

(14) $$\begin{array}{r} {\hat{H}}_{\text{pl}}=\underset{w}{\sum }\hbar \omega_{w}{\hat{b}}_{w}^{†}{\hat{b}}_{w}+\frac{1}{2}. \end{array}$$

The plasmonic Hamiltonian may be written in this way when initially ignoring losses; otherwise the plasmonic modes are ill-defined (Waks and Sridharan, 2010; Finazzi and Ciccacci, 2012). We account for the decay by using complex energies ω_*w*_ + iγ_*w*_ in the energy denominators of Equations (1–3) as is common practice within the microscopic theory of Raman scattering (Long, 2002; Yu and Cardona, 2010). The Hamiltonian in Equation (14) can also be applied for oligomers or arrays of plasmonic nanoparticles (Brandstetter-Kunc et al., 2015, 2016; Lamowski et al., 2018). In this case ${\hat{b}}_{w}$ corresponds to the operator of the hybridized plasmon modes *w* of the coupled nanoparticles and is given by a Bogoliubov transformation of the single nanoparticle operators.

### 3.2. Plasmon-Photon Interaction

In the presence of an external light field, the plasmonic Hamiltonian in Equation (10) has to be modified by the Peierl's substitution ${\hat{\Pi }}_{w}\to {\hat{\Pi }}_{w}+e{\hat{A}}_{w}$ which leads to the minimal coupling Hamiltonian

(15) $$\begin{array}{r} {\hat{H}}_{\text{pl}-\text{pt}}=\frac{eNV_{\text{p}}}{m}\underset{w}{\sum }{\hat{\Pi }}_{w}{\hat{A}}_{w}. \end{array}$$

*Â*_*w*_ is a projection of the vector potential ${\hat{\text{A}}}_{\text{pt}}$ of the external light field onto a plasmonic mode *w*. It can be calculated with a volume integral approach as Finazzi and Ciccacci (2012)

(16) $$\begin{array}{r} {\hat{A}}_{w}=C_{\text{LF}}\int_{V_{\text{p}}}\text{d}{\text{r}}^{\prime }\;{\hat{\text{A}}}_{\text{pt}}\left({\text{r}}^{\prime },\omega_{w}\right)\cdot {\text{q}}_{w}\left({\text{r}}^{\prime }\right), \end{array}$$

where **q**_*w*_(**r**) is the eigenvector of a plasmon mode *w* which has to fulfill the normalization condition (Yu et al., 2017)

(17) $$\begin{array}{r} \int_{V_{p}}d\text{r}\int_{V_{p}}d{\text{r}}^{\prime }{\text{q}}_{w}\left(\text{r}\right)\cdot {\text{q}}_{w^{\prime }}\left({\text{r}}^{\prime }\right)=\delta_{w,w^{\prime }}. \end{array}$$

*C*_LF_ is a local field correction factor which accounts for the difference between the microscopic light field that couples to the plasmonic mode **q**_*w*_(**r**) and the incident macroscopic light field ${\hat{\text{A}}}_{\text{pt}}$ (Onsager, 1936; de Vries and Lagendijk, 1998; Dolgaleva and Boyd, 2012). For a single nanoparticle it is given by

(18) $$\begin{array}{r} C_{\text{LF}}=\frac{\epsilon_{m}\omega_{w}^{2}}{L\omega_{p}^{2}}, \end{array}$$

where *L* is a depolarization factor that accounts for the shape of the nanoparticle, $\omega_{p}=\sqrt{Ne^{2}/\epsilon_{0}m}$ is the plasma frequency of the metal and ϵ_*m*_ is the dielectric constant of the surrounding medium; see Appendix for details. We note that this correction factor was not included in Mueller et al. (2016) which lead to an overestimation of the SERS enhancement calculated from the coupling matrix elements.

To derive an explicit expression for the photon-plasmon interaction Hamiltonian, we express the vector potential of the light field with second quantization as Ho and Kumar (1993); Loudon (2000)

(19) $$\begin{array}{r} {\hat{\text{A}}}_{\text{pt}}\left(\text{r},\omega_{\text{pt}}\right)={\tilde{A}}_{\text{pt}}\varepsilon_{\text{pt}}\left({\hat{a}}_{\text{pt}}{\text{e}}^{\text{i}{\text{k}}_{\text{pt}}\cdot \text{r}}+{\hat{a}}_{\text{pt}}^{†}{\text{e}}^{-\text{i}{\text{k}}_{\text{pt}}\cdot \text{r}}\right), \end{array}$$

where we considered for simplicity only one wavevector **k**_pt_ and polarization **ε**_pt_. The amplitude of the light field is ${\tilde{A}}_{\text{pt}}=\sqrt{\hbar /2\omega_{\text{pt}}V_{\text{R}}\epsilon_{0}\epsilon_{\text{m}}}$ with a normalization volume *V*_R_ and the frequency of the light field ω_pt_. By using Equations (15), (12), (16), and (19) and dropping the counter-rotating terms the interaction Hamiltonian is given by

(20) $$\begin{array}{r} {\hat{H}}_{\text{pl}-\text{pt}}=\text{i}e\hbar \sqrt{\frac{NV_{\text{p}}}{4mV_{\text{R}}\epsilon_{0}\epsilon_{m}}}\underset{w}{\sum }\left(\varepsilon_{w}^{\text{pt}}{\hat{a}}_{\text{pt}}{\hat{b}}_{w}^{†}-{\left(\varepsilon_{w}^{\text{pt}}\right)}^{*}{\hat{a}}_{\text{pt}}^{†}{\hat{b}}_{w}\right), \end{array}$$

where

(21) $$\begin{array}{r} \varepsilon_{w}^{\text{pt}}=C_{\text{LF}}\int_{V_{\text{p}}}\text{d}{\text{r}}^{\prime }\varepsilon_{\text{pt}}\cdot {\text{q}}_{w}\left({\text{r}}^{\prime }\right){\text{e}}^{\text{i}{\text{k}}_{\text{pt}}\cdot {\text{r}}^{\prime }} \end{array}$$

is a factor that gives the selection rules for the interaction of light with plasmonic modes *w*.

To discuss the applicability of the interaction Hamiltonian in Equation (20) we will first calculate the optical absorption cross section of plasmonic nanoparticles and draw a comparison to experimental data.

The absorption of a plasmon mode with frequency ω_pl_ and spectral width 2γ_pl_ is given by the cross section

(22) $$\begin{array}{r} \sigma_{\text{abs}}\left(\omega_{\text{L}}\right)=\frac{2V_{\text{R}}\sqrt{\epsilon_{m}}}{cn_{\text{pt}}\hbar^{2}}|M_{\text{pl}-\text{pt}}{|}^{2}\frac{\gamma_{\text{pl}}}{{\left(\omega_{\text{L}}-\omega_{\text{pl}}\right)}^{2}+\gamma_{\text{pl}}^{2}}, \end{array}$$

with the coupling matrix element

(23) $$\begin{array}{r} M_{\text{pl}-\text{pt}}=\langle 1_{\text{pl}},n_{\text{pt}}-1|H_{\text{pl}-\text{pt}}|0_{\text{pl}},n_{\text{pt}}\rangle . \end{array}$$

The absorption cross section is obtained by dividing the plasmon excitation rate (Fermi Golden Rule)

(24) $$\begin{array}{r} \Gamma_{\text{pl}}=\frac{2}{\hbar^{2}}|M_{\text{pl}-\text{pt}}{|}^{2}\frac{\gamma_{\text{pl}}}{{\left(\omega_{\text{L}}-\omega_{\text{pl}}\right)}^{2}+\gamma_{\text{pl}}^{2}} \end{array}$$

by the photon flux of the incident light field $|\langle n_{\text{pt}}|\hat{\text{S}}|n_{\text{pt}}\rangle |=n_{\text{pt}}c/V_{\text{R}}\sqrt{\epsilon_{m}}$, where $\hat{\text{S}}$ is the Poynting vector. We assumed a Lorentzian line shape of the plasmon resonance as in the SERS scattering amplitudes in Equations (1)–(3).

The plasmonic properties of small nanoparticles are well described by the point dipole approximation and the plasmon eigenvector of a dipole mode is given by

(25) $$\begin{array}{r} {\text{q}}_{\text{pl}}\left(\text{r}\right)=\varepsilon_{\text{pl}}\delta \left(\text{r}-{\text{r}}_{\text{pl}}\right), \end{array}$$

where **ε**_pl_ is the polarization and **r**_pl_ is the position of the particle center. For larger nanoparticles the eigenvectors may be calculated with Mie theory as we demonstrated in Mueller et al. (2016). Using Equations (18) and (20)–(25) the absorption cross section of a plasmonic nanoparticle can be expressed as

(26) $$\begin{array}{r} \sigma_{\text{abs}}\left(\omega_{\text{L}}\right)=\frac{\epsilon_{m}^{3/2}V_{\text{p}}\omega_{\text{pl}}^{4}}{2cL^{2}\omega_{p}^{2}}\frac{\gamma_{\text{pl}}}{{\left(\omega_{\text{L}}-\omega_{\text{pl}}\right)}^{2}+\gamma_{\text{pl}}^{2}}. \end{array}$$

**Figure 3:**
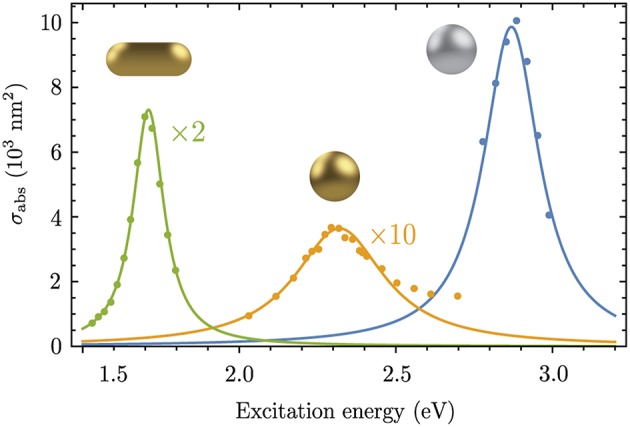
Experimental and calculated (Equation 26) optical absorption cross sections for a gold nanorod (green, Lombardi et al., 2012), a gold nanosphere (orange, Billaud et al., 2007) and a silver nanosphere (blue, Muskens et al., 2006). The nanoparticle volume *V*_p_ was taken from the experimental works. The following parameters were used to reproduce the experimental spectra: ℏω_pl_ = 1.71 eV, ℏγ_pl_ = 60 meV, ϵ_*m*_ = 2.02 and *L* = 0.12 for the Au nanorod; ℏω_pl_ = 2.32 eV, ℏγ_pl_ = 175 meV, ϵ_*m*_ = 1.96 and *L* = 1/3 for the Au nanosphere; ℏω_pl_ = 2.87 eV, ℏγ_pl_ = 108 meV, ϵ_*m*_ = 2.31 and *L* = 1/3 for the Ag nanosphere.

The absorption cross section as a function of energy can be measured with optical modulation spectroscopy (Crut et al., 2014). In Figure 3 we compare the absorption cross section from Equation (26) with experimental data for spherical silver and gold nanoparticles and a gold nanorod from Lombardi et al. (2012), Billaud et al. (2007), and Muskens et al. (2006). By using ω_pl_ and γ_pl_ as fitting parameters and the particle volume *V*_p_ measured in experiments we obtain perfect agreement with the magnitude and energy dependence of the experimental cross sections. In the case of the gold nanosphere the theory underestimates the cross section for energies larger than 2.5 eV. The asymmetry in the excitation energy dependence appears because of interband transitions which is not captured by the Lorentzian line profile in Equation (26). On the other hand, the excellent agreement of the magnitudes shows that the local field correction factor in Equation (18) correctly accounts for the optical properties of gold and silver at the energies ℏω_pl_ of the localized surface plasmon resonances. The plasmon-photon interaction Hamiltonian in Equation (20) therefore gives the correct oscillator strength and will be used below to calculate the SERS enhancement for a molecule close to a plasmonic nanoparticle.

### 3.3. Plasmon-Molecule Interaction

We consider the coupling of the plasmonic nanostructure to a molecule with vibronic states as illustrated in Figure 1A. We assume that the interaction is of dipolar nature and use the interaction Hamiltonian (Waks and Sridharan, 2010)

(27) $$\begin{array}{r} {\hat{H}}_{\text{pl}-\text{vib}}=-\hat{\mu }\cdot {\hat{\text{E}}}_{\text{LSP}}\left(\text{r}\right), \end{array}$$

where ${\hat{\text{E}}}_{\text{LSP}}\left(\text{r}\right)$ is the electric field generated by the plasmonic nanostructure and

(28) $$\hat{\mu }=\sum_{m,n}\mu_{mn}|n\rangle \langle m|$$

is a generic transition operator for the dipole transitions of the molecule. The matrix element **μ**_*mn*_ gives the transition probability from a vibronic state |*m*〉 to a state |*n*〉. The point dipole approximation for the molecule is justified as long as the plasmonic near field ${\hat{\text{E}}}_{\text{LSP}}\left(\text{r}\right)$ is approximately constant over the size of the molecule, which is a good approximation for metal-molecule distances much larger than the size of the molecule. For smaller distances, surface roughness and atomic scale protrusions of the metallic nanostructure become important which can be modeled by including field-dependent terms to the permanent molecular dipole in Equation (28) or by a quantum-mechanical modeling of the molecular states (Ayars et al., 2000; Jensen et al., 2008; Neuman et al., 2018).

We calculate the electric near field **E**_LSP_(**r**) = −∇_**r**_*ϕ*_pl_(**r**) of the plasmonic nanostructure within the electrostatic approximation as the gradient of the scalar potential

(29) $$\begin{array}{r} \phi_{\text{pl}}\left(\text{r}\right)=-\frac{1}{\epsilon_{0}\epsilon_{m}}\int_{V_{\text{p}}}\text{d}{\text{r}}^{\prime }\rho \left({\text{r}}^{\prime }\right)G_{0}\left(\text{r},{\text{r}}^{\prime }\right). \end{array}$$

$G_{0}\left(\text{r},{\text{r}}^{\prime }\right)$ is a Green function that gives the field distribution outside the plasmonic nanoparticle and ρ(**r**) is the electric charge density (Novotny and Hecht, 2012). In order to obtain a quantized expression for the plasmonic near field we express the charge density $\rho \left(\text{r}\right)=-NeC_{\text{LF}}\nabla_{\text{r}}\cdot \text{s}\left(\text{r}\right)$ in terms of a microscopic displacement **s**(**r**) of the charges inside the nanoparticle. It was shown in Finazzi and Ciccacci (2012) that a projection of the charge displacement onto the plasmonic eigenvectors can be substituted by the generalized position operator as

(30) $$\int_{V_{\text{p}}}\text{d}r^{\prime }\;s(r^{\prime })\cdot q_{w}(r^{\prime })\to {\hat{\Psi }}_{w}.$$

By using the normalization condition for the plasmonic eigenvectors in Equation (17), the plasmonic near field can be written as

(31) $$\begin{array}{r} {\hat{\text{E}}}_{w}\left(\text{r}\right)=-\frac{NeV_{p}}{\epsilon_{0}\epsilon_{m}}{\hat{\text{Ψ}}}_{w}{\text{G}}_{w}\left(\text{r}\right), \end{array}$$

with

(32) $$\begin{array}{r} {\text{G}}_{w}\left(\text{r}\right)=C_{\text{LF}}\nabla_{\text{r}}\int_{V_{\text{p}}}\text{d}{\text{r}}^{\prime }{\text{q}}_{w}\left({\text{r}}^{\prime }\right)\cdot \nabla_{{\text{r}}^{\prime }}G_{0}\left(\text{r},{\text{r}}^{\prime }\right). \end{array}$$

The position dependence and polarization of the plasmonic near field is entirely contained in **G**_*w*_(**r**) and ${\hat{\text{Ψ}}}_{w}$ contains plasmonic creation and annihilation operators; see Equation (11). Based on Equations (27) and (31) we obtain

(33) $$\begin{array}{r} H_{\text{pl}-\text{vib}}=\underset{w}{\sum }\frac{e}{\epsilon_{0}\epsilon_{m}}\sqrt{\frac{\hbar NV_{p}}{2m\omega_{w}}}\left({\hat{b}}_{w}^{†}+{\hat{b}}_{w}\right)\hat{\mu }\cdot {\text{G}}_{w}\left(\text{r}\right) \end{array}$$

for the plasmon-molecule interaction Hamiltonian.

Finally we also consider the direct coupling of the incident light to the molecular transition dipole, which is described by the interaction Hamiltonian ${\hat{H}}_{\text{pt}-\text{vib}}=-\hat{\mu }\cdot {\hat{\text{E}}}_{\text{pt}}\left(\text{r}\right)$. From Equation (19) and ${\hat{\text{E}}}_{\text{pt}}\left(\text{r}\right)=-\partial {\hat{\text{A}}}_{\text{pt}}\left(\text{r}\right)/\partial t$ we obtain the explicit expression

(34) $$\begin{array}{r} {\hat{H}}_{\text{pt}-\text{vib}}=-\text{i}\sqrt{\frac{\hbar \omega_{\text{pt}}}{2V_{\text{R}}\epsilon_{0}\epsilon_{\text{m}}}}\hat{\mu }\cdot \varepsilon_{\text{pt}}\left({\hat{a}}_{\text{pt}}{\text{e}}^{\text{i}{\text{k}}_{\text{pt}}\cdot \text{r}}-{\hat{a}}_{\text{pt}}^{†}{\text{e}}^{-\text{i}{\text{k}}_{\text{pt}}\cdot \text{r}}\right). \end{array}$$

## 4. SERS Enhancement by a Silver Nanosphere

In order to discuss the magnitude and excitation energy dependence of the plasmonic enhancement we calculate the enhancement of the SERS cross section for a molecule next to a silver nanoparticle. We consider a SERS experiment in which the same molecule is used as SERS analyte and reference and the molecular resonance is not perturbed by the metal surface; see e.g., Mueller et al. (2017b). In this case the SERS enhancement is given by the plasmonic enhancement factor in Equation (7). From the analytic expressions for the interaction Hamiltonians above we calculate the coupling factors as.

(35) $$\begin{array}{r} {\tilde{M}}_{1}={\tilde{M}}_{2}=-\frac{\hbar \omega_{p}^{2}V_{\text{p}}}{2\epsilon_{m}\omega_{\text{pl}}}\varepsilon_{\text{pl}}^{\text{pt}}\frac{{\text{e}}_{\text{mol}}\;\cdot \;{\text{G}}_{\text{pl}}\left(\text{r}\right)}{{\text{e}}_{\text{mol}}\;\cdot \;\varepsilon_{\text{pt}}}, \end{array}$$

where “pl” refers to the dipolar plasmon resonance of the silver nanoparticle. **e**_mol_ is a unit vector along the transition dipole of the molecule. The coupling factors ${\tilde{M}}_{1}$ and ${\tilde{M}}_{2}$ are only equal for a Raman process where **μ**_*gi*_ ∥ **μ**_*if*_. For the more general case of a Raman tensor with off-diagonal elements **e**_mol_ may differ in ${\tilde{M}}_{1}$ and ${\tilde{M}}_{2}$.

In the following, we consider the spherical silver nanoparticle for which we calculated the absorption cross section in Figure 3 with radius *r*_NP_ = 15.5 nm. As this nanoparticle is small compared to the wavelength of the incident light (350–500 nm), we calculate the plasmonic eigenvector of the dipole mode with the point dipole approximation; see Equation (25). That way we obtain $\varepsilon_{\text{pl}}^{\text{pt}}=C_{\text{LF}}$ and

(36) $$\begin{array}{r} {\text{G}}_{\text{pl}}\left(\text{r}\right)=\frac{C_{\text{LF}}}{4\pi |\text{r}{|}^{3}}\left(3\frac{\left(\varepsilon_{\text{pt}}\cdot \text{r}\right)\text{r}}{|\text{r}{|}^{2}}-\varepsilon_{\text{pt}}\right). \end{array}$$

The coupling factors ${\tilde{M}}_{1}$ and ${\tilde{M}}_{2}$ are, within the approximations made here, real valued quantities and take negative values for the places of strongest field enhancement. This nicely agrees with the assumptions that were made to explain the experimental SERS resonance profiles in Figure 2.

**Figure 4:**
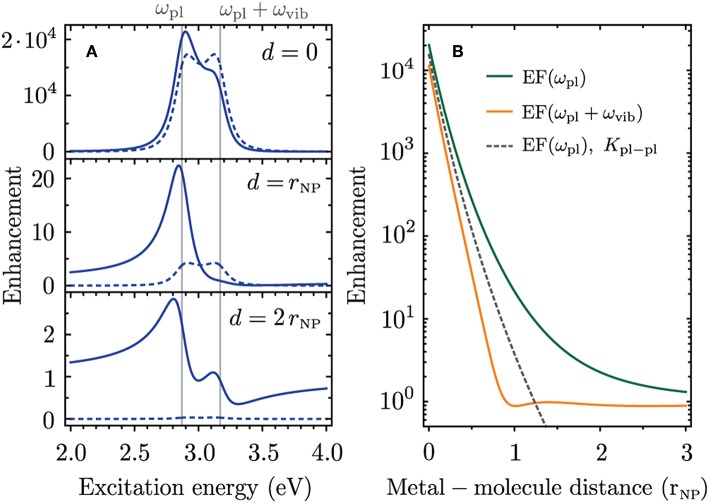
Plasmonic enhancement of the Raman cross section for a molecule close to a silver nanoparticle with properties similar to those in Figure 3, i.e., an Ag nanosphere with radius *r*_NP_ = 15.5 nm, ℏω_pl_ = 2.87 eV, ℏγ_pl_ = 108 meV and ϵ_m_ = 2.31. We place the Ag nanosphere center at the origin, consider **ε**_pl_, **e**_mol_ ∥ *x* and move the molecule along the *x* axis. **(A)** Excitation energy dependence of the enhancement for three distances *d* of the molecule to the metal surface. **(B)** Enhancement at ω_pl_ (incoming plasmonic Raman resonance, green) and ω_pl_ + ω_vib_ (outgoing plasmonic Raman resonance, orange) as a function of metal-molecule distance. Dashed lines show the enhancement when only *K*_pl−pl_ is used to calculate the enhancement factor.

In Figure 4 we calculate the plasmonic SERS enhancement for a molecule close to the silver nanoparticle. We assume a molecular transition dipole parallel to the polarization of the incident light field and to the plasmonic near field. This configuration leads to the largest enhancement. The excitation energy dependence of the SERS enhancement is plotted in Figure 4 for different distances of the molecule to the silver nanoparticle. We consider a Raman shift of ℏω_vib_ = 0.3 eV which is larger than the spectral width 2γ_pl_ ≈ 0.2 eV of the plasmon resonance. In this case the incoming and outgoing Raman resonances are visible as distinct and overlapping peaks in the SERS enhancement. When the molecular dipole is placed on the surface of the silver nanoparticle the Raman cross section is enhanced by a factor of 2 × 10^4^ (Figure 4A, *d* = 0, solid line). The incoming and outgoing plasmonic Raman resonances provide almost the same enhancement with a slight dominance of the incoming Raman resonance. The resonance profile looks strikingly different when the molecule is moved away from the metal surface. When the metal-molecule distance equals the nanoparticle radius (*d* = *r*_NP_) the enhancement occurs only at the incoming plasmonic Raman resonance with a factor of ~20 while the outgoing plasmonic Raman resonance is entirely missing. For an even larger distance of *d* = 2*r*_NP_ the incoming and outgoing resonances obtain a Fano-like profile and only the incoming resonance provides a modest enhancement of 2.8. In the following we refer to these three cases as the regimes of strong, intermediate and weak plasmonic enhancement.

The difference in enhancement at the incoming and outgoing plasmonic Raman resonances occurs because of interference between the scattering channels that are illustrated as Feynman diagrams in Figures 1B,C (i)–(iii). These scattering processes have the same final state, i.e., a molecular vibration $\omega_{f}^{\text{vib}}$ is excited and the Raman-scattered light $\omega_{\text{L}}-\omega_{f}^{\text{vib}}$ is emitted. The corresponding Raman amplitudes are therefore summed in Equation (6) before calculating the absolute square and the different terms can add constructively or destructively. When only considering the process in Figure 1B, where the incoming- and the Raman-scattered light couple to the plasmon, both Raman resonances provide the same enhancement (dashed lines in Figure 4A). This process is the dominant contribution in the regime of strong plasmonic enhancement, i.e., for *d* ≈ *r*_NP_. In the intermediate enhancement regime the scattering processes in Figure 1C (i) and (ii), where the plasmon resonance enhances either the incoming or the Raman-scattered light, have to be taken into account to explain the SERS resonance profile. The Raman amplitudes of the different scattering processes add constructively at the incoming plasmonic Raman resonance and cancel each other at the outgoing plasmonic Raman resonance. In the weak enhancement regime the Raman process without plasmonic enhancement (Figure 1C (iii)] becomes important as the enhancement is close to one. The contribution from the process in Figure 1B is negligible in this case.

In Figure 4B we plot the enhancement at the energies of the incoming and Raman-scattered light as a function of metal-molecule distance. The enhancement from the scattering process in Figure 1B is plotted as a dashed line for comparison. A constructive interference at the incoming plasmonic Raman resonance and destructive interference at the outgoing plasmonic Raman resonance occurs for all metal-molecule distances. The difference in enhancement is largest around *d* = 2*r*_NP_. More generally the effect of interference between different scattering channels is most pronounced when ${\tilde{M}}_{1}={\tilde{M}}_{2}=-\hbar \omega_{\text{vib}}$, which corresponds to the intermediate enhancement regime with enhancement factors of 10^1^ − 10^2^. The effect is clearly irrelevant for single-molecule SERS which requires enhancement factors on the order of 10^7^ − 10^9^ (Ru and Etchegoin, 2009). On the other hand, the intermediate enhancement regime becomes important when a spatially extended film of molecules or a two-dimensional material is coupled to a plasmonic nanostructure (McFarland et al., 2005; Heeg et al., 2013). Furthermore, the enhancement factors in tip-enhanced Raman scattering (TERS) are typically in the intermediate enhancement regime (Beams et al., 2014; Wang et al., 2017). The constructive interference between different scattering pathways can strongly increase the enhancement at a specific excitation energy making TERS sensitive to near-field coupling.

## 5. Comparison to Theory of Electromagnetic Enhancement

The plasmonic enhancement in SERS is commonly estimated by a theory that treats that plasmonic nanostructure as a nanoscale antenna which increases the local light intensity (Ru and Etchegoin, 2009; Ding et al., 2017). The enhancement of the Raman intensity for a molecule at position **r** is given by

(37) $$\begin{array}{r} \text{E}{\text{F}}_{\text{EM}}\left(\text{r},\omega \right)=\frac{|{\text{E}}_{\text{loc}}\left(\text{r},\omega \right){|}^{2}}{|{\text{E}}_{0}\left(\text{r},\omega \right){|}^{2}}\frac{|{\text{E}}_{\text{loc}}\left(\text{r},\omega -\omega_{\text{vib}}\right){|}^{2}}{|{\text{E}}_{0}\left(\text{r},\omega -\omega_{\text{vib}}\right){|}^{2}}, \end{array}$$

where **E**_loc_ is the local electric field amplitude and **E**_0_ the amplitude of the incoming light field without enhancement. This theory of electromagnetic (EM) enhancement is a powerful tool for the design of plasmonic nanostructures with strong SERS enhancement (Ding et al., 2017). The local electric field enhancement can be calculated for complicated nanostructure geometries with numerical techniques (Solís et al., 2014). On the other hand, a precise knowledge of the nanostructure geometry is required to interpret the outcome of a SERS experiment and molecular resonances are not included in the enhancement factor. Our approach benefits from microscopic insight into the scattering processes underlying SERS and can be used to extract the spectral properties of the plasmonic and molecular resonances from SERS profiles. In the following we will show that both theories predict the same plasmonic enhancement and the enhancement factor in Equation (37) can be rewritten into an expression that is formally equivalent to Equation (7).

An important aspect that is disregarded when writing the electromagnetic enhancement factor as in Equation (37) is that only the electric field component along the molecular transition dipole contributes to the enhancement. We therefore project the local electric field vectors onto the transition dipoles **μ**_*gi*_ and **μ**_*if*_ that are relevant for the Raman process (see Figure 1A). By expressing the local electric field **E**_loc_ as the sum of the plasmonic near field **E**_pl_ and the incident light field **E**_0_ we obtain

(38) $$\begin{array}{r} \text{E}{\text{F}}_{\text{EM}}\left(\text{r},\omega \right)=|1+f_{\text{in}}\left(\text{r},\omega \right){|}^{2}|1+f_{\text{out}}\left(\text{r},\omega \right){|}^{2}, \end{array}$$

with

(39) $$\begin{array}{r} f_{\text{in}}\left(\text{r},\omega \right)=\frac{\mu_{gi}\;\cdot \;{\text{E}}_{\text{pl}}\left(\text{r},\omega \right)}{\mu_{gi}\;\cdot \;{\text{E}}_{0}\left(\text{r},\omega \right)} \end{array}$$

and

(40) $$\begin{array}{r} f_{\text{out}}\left(\text{r},\omega \right)=\frac{\mu_{if}\;\cdot \;{\text{E}}_{\text{pl}}\left(\text{r},\omega -\omega_{\text{vib}}\right)}{\mu_{if}\;\cdot \;{\text{E}}_{0}\left(\text{r},\omega -\omega_{\text{vib}}\right)}. \end{array}$$

This enhancement factor can be rewritten as

(41) $$\begin{array}{r} \text{E}{\text{F}}_{\text{EM}}\left(\text{r},\omega \right)=|f_{\text{in}}\left(\text{r},\omega \right)f_{\text{out}}\left(\text{r},\omega \right)+f_{\text{in}}\left(\text{r},\omega \right)+f_{\text{out}}\left(\text{r},\omega \right)+1{|}^{2}, \end{array}$$

which is formally equivalent with the plasmonic enhancement factor from our microscopic approach in Equation (7). The four terms can be identified with the scattering processes in Figures 1B,C (i)–(iii).

In order to compare the enhancement predicted by the two theories we repeat the model calculations in Figure 4 based on the enhancement factor in Equation (41). Within the quasi-static approximation the plasmonic near field of the silver nanoparticle is

(42) $$\begin{array}{r} {\text{E}}_{\text{pl}}\left(\text{r},\omega \right)=\frac{1}{4\pi \epsilon_{0}|\text{r}{|}^{3}}\left(3\frac{\left({\text{p}}_{\text{pl}}\left(\text{r},\omega \right)\;\cdot \;\text{r}\right)\text{r}}{|\text{r}{|}^{2}}-{\text{p}}_{\text{pl}}\left(\text{r},\omega \right)\right), \end{array}$$

with the plasmonic dipole moment

(43) $$\begin{array}{r} {\text{p}}_{\text{pl}}\left(\text{r},\omega \right)=\alpha \left(\omega \right)E_{0}\varepsilon_{\text{pt}}\delta \left(\text{r}\right), \end{array}$$

and the polarizability of the nanosphere

(44) $$\begin{array}{r} \alpha \left(\omega \right)=4\pi \epsilon_{0}\epsilon_{\text{m}}r_{\text{NP}}^{3}\frac{\epsilon_{\text{Ag}}\left(\omega \right)\;-\;\epsilon_{\text{m}}}{\epsilon_{\text{Ag}}\left(\omega \right)\;+\;2\epsilon_{\text{m}}}. \end{array}$$

**Figure 5:**
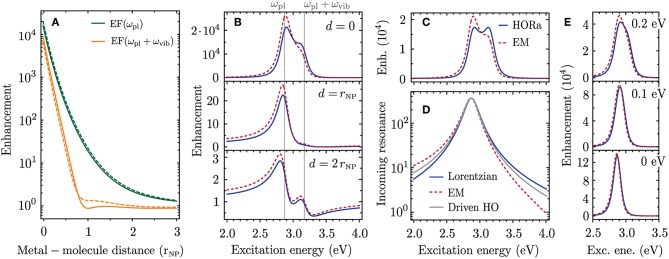
Comparison of enhancement factors from higher-order Raman approach (HORa, solid lines) and electromagnetic enhancement theory (EM, dashed lines). The parameters are the same as in Figure 4. **(A)** Enhancement at the incoming- and outgoing plasmonic Raman resonance as a function of metal-molecule distance. **(B)** Excitation energy dependence of the enhancement for three metal-molecule distances. **(C)** Enhancement for *d* = 0 when only considering the scattering process where the incoming- and Raman-scattered light couples to the plasmon. **(D)** Enhancement provided by the incoming plasmonic Raman resonance for a Lorentzian line profile as in the HORa approach (blue solid line), for the EM enhancement theory (red dashed line) and for the resonance of a driven harmonic oscillator (gray solid line). **(E)** Enhancement profiles for different Raman shifts given in the insets.

We use a Drude model for the dielectric function of silver ϵ_Ag_(ω) from Yang et al. (2015) and include a phenomenological surface broadening term to reproduce the experimentally determined spectral width of the plasmon resonance (see Muskens et al., 2006). In Figure 5A we compare the enhancement factors at the incoming and outgoing plasmonic Raman resonances as a function of metal-molecule distance. There is good agreement of the enhancement predicted by our microscopic approach (HORa, solid lines) and the electromagnetic enhancement model (EM, dashed lines). The weaker enhancement at the outgoing resonance is also predicted by the electromagnetic enhancement factor. When calculating the resonance profiles for different metal-molecule distances we obtain overall good agreement between both theories for all enhancement regimes (Figure 5B).

Upon a closer look it appears that the difference in enhancement at the incoming and outgoing plasmonic Raman resonances is even more pronounced for the EM enhancement model. To explain this small discrepancy we calculate the enhancement profiles for the case when only the scattering process in Figure 1B is considered; see Figure 5C. While the HORa theory predicts in this case two overlapping Raman resonances of equal intensity, the enhancement profile from the EM enhancement theory remains asymmetric. This asymmetry cannot be explained by interference between different scattering channels but is attributed to an intrinsic asymmetry of the plasmon resonance; see Figure 5D. It is only visible on a logarithmic scale and negligible in the absorption spectra in Figure 3. However, when multiplying two resonances that are spectrally displaced by the Raman shift, as is the case in Figure 5C, the asymmetry becomes important.

As our microscopic model is based on higher-order perturbation theory the plasmonic and molecular resonances have a symmetric Lorentzian line profile (blue line in Figure 5D). The asymmetry of the plasmonic resonance from the EM model arises for two reasons. First, when considering the localized surface plasmon as a driven and damped harmonic oscillator the Lorentzian energy denominators in Equation (7) have to be replaced by Zuloaga and Nordlander (2011)

(45) $$\begin{array}{r} \frac{1}{\omega -\omega_{\text{pl}}+\text{i}\gamma_{\text{pl}}}\to \frac{2\omega_{\text{pl}}}{\omega^{2}-\omega_{\text{pl}}^{2}+2\text{i}\gamma_{\text{pl}}\omega }, \end{array}$$

which leads to a slight asymmetry of the plasmon resonance (gray line in Figure 5D). Second, the depolarization by the bound electrons which we modeled with a local field correction factor *C*_LF_ is wavelength dependent and increases toward shorter wavelength. In this work we derived an analytic expression that is strictly only valid at the plasmon resonance [Equation (18)]. How to incorporate the wavelength dependent damping and depolarization when quantizing a localized surface plasmon oscillation is a matter of ongoing research and will be key to fully capture the energy dependence of the SERS enhancement. On the other hand, the differences in the enhancement profiles from EM and HORa only occur when the Raman shift is larger than the spectral width of the plasmon. For smaller Raman shifts the incoming and outgoing plasmonic Raman resonances cannot be spectrally resolved and there is excellent agreement between the enhancement profiles from both theories (Figure 5E).

## 6. Conclusions

In conclusion we presented a microscopic model of the plasmonic enhancement mechanism in SERS which is based on perturbation theory. The main idea of our approach is to treat the plasmonic excitation as an integral part of the Raman scattering process. This leads to a description of SERS as higher-order Raman scattering. We derived analytic expressions for the Raman scattering amplitudes that can be used to study the interplay of plasmonic and molecular resonances or the interference between different scattering channels. As the properties of the plasmonic and molecular resonances, such as frequency, spectral width and oscillator strength, appear as explicit parameters in the theoretical framework, it can be used for the interpretation of experimental data.

Based on a quantization of the localized surface plasmon we derived analytic expressions for all coupling matrix elements that describe the oscillator strength of the material excitations and lead to selection rules for SERS. We demonstrated that a local field correction factor must be included in the quantization model of the plasmon in order to reproduce the experimental absorption cross sections of plasmonic nanoparticles. This was not considered in our previous work (Mueller et al., 2016) and lead to an overestimation of the SERS enhancement factor. With model calculations for a molecule close to a silver nanoparticle we showed that interference between different scattering channels can strongly affect the excitation energy dependence of the SERS enhancement. This effect is most relevant in the intermediate enhancement regime with enhancement factors of 10−10^3^ and is therefore particularly important when plasmonic nanostructures are coupled to spatially extended materials. We showed that the plasmonic enhancement obtained from our microscopic approach is overall in good agreement with that predicted by the commonly used theory of EM enhancement. While the EM enhancement theory is a powerful tool to design SERS substrates with strong plasmonic enhancement our approach gives microscopic insight, serves well in fitting experimental data, and can be used to study the interplay of different scattering channels underlying SERS.

## Data Availability

All datasets generated for this study are included in the manuscript and/or the supplementary files.

## Author Contributions

Both authors contributed in multiple ways to the research presented in this manuscript.

### Conflict of Interest Statement

The authors declare that the research was conducted in the absence of any commercial or financial relationships that could be construed as a potential conflict of interest.

## Appendix

### Local Field Correction Factor

As demonstrated above, a local field correction factor *C*_LF_ must be included in our microscopic theory to reproduce the experimental absorption cross sections of plasmonic nanoparticles (see Figure 3). In this appendix we will derive the general expression for *C*_LF_ in Equation (18).

Due to the normalization of the plasmon eigenvectors (Equation 17) all prefactors that accounted for the coupling to an external light field are not included. The plasmon eigenvector **q**_*w*_ describes the microscopic oscillation of free charges inside the nanoparticle. The incident light field is described by a macroscopic amplitude ${\hat{\text{A}}}_{\text{pt}}\left(\text{r}\right)$ which differs from the microscopic amplitude inside the nanoparticle because of a depolarization of the incident light field by the bound electrons of the metal. This can be modeled by considering a plasmonic point dipole inside a spherical nanoparticle with a dielectric function ϵ_*b*_ that accounts for the effect of the bound charges and interband transitions (Lamowski et al., 2018). The amplitude of the light field inside the nanoparticle is given within the quasi-static approximation by Bohren and Huffman (1998)

(A1) $$\begin{array}{r} {\text{A}}_{\text{pt}}^{\text{int}}=\frac{3\epsilon_{m}}{\epsilon_{b}+2\epsilon_{m}}{\text{A}}_{\text{pt}}. \end{array}$$

The factor 3ϵ_*m*_/(ϵ_*b*_ + 2ϵ_*m*_) is known as a local field correction factor and might take different forms depending on the considered geometry (Onsager, 1936; de Vries and Lagendijk, 1998; Dolgaleva and Boyd, 2012). It must be also included in ${\hat{H}}_{\text{pl}-\text{vib}}$ when calculating the quantized electric field of the plasmon in Equations (31) and (32).

To obtain a more general expression we consider a nanoparticle with an ellipsoidal shape. Using a Drude model for the electromagnetic response of the metal, the dipolar plasmon resonance occurs at Bohren and Huffman (1998) and Ru and Etchegoin (2009)

(A2) $$\begin{array}{r} \omega_{\text{pl}}=\frac{\omega_{\text{p}}}{\sqrt{\epsilon_{b}+\epsilon_{m}\left(1/L-1\right)}}, \end{array}$$

where *L* is a shape-dependend depolarization factor which is 1/3 for a nanosphere (for explicit expressions see Bohren and Huffman, 1998; Ru and Etchegoin, 2009). The amplitude of the incident light field inside the nanoparticle is given by

(A3) $$\begin{array}{r} {\text{A}}_{\text{pt}}^{\text{int}}=\frac{\epsilon_{m}}{L\epsilon_{b}+\epsilon_{m}\left(1-L\right)}{\text{A}}_{\text{pt}}. \end{array}$$

Using Equation (2) we obtain the local field correction factor

(A4) $$\begin{array}{r} C_{\text{LF}}=\frac{\epsilon_{m}\omega_{\text{pl}}^{2}}{L\omega_{p}^{2}}. \end{array}$$

As this factor only contains the bulk plasma frequency of the metal ω_p_, the dielectric constant of the environment ϵ_*m*_ and the plasmon frequency ω_pl_ it can be used for any dipolar plasmon mode of a single nanoparticle.
